# The role of cysteine peptidases in coronavirus cell entry and replication: The therapeutic potential of cathepsin inhibitors

**DOI:** 10.1371/journal.ppat.1009013

**Published:** 2020-11-02

**Authors:** Anja Pišlar, Ana Mitrović, Jerica Sabotič, Urša Pečar Fonović, Milica Perišić Nanut, Tanja Jakoš, Emanuela Senjor, Janko Kos

**Affiliations:** 1 Department of Pharmaceutical Biology, Faculty of Pharmacy, University of Ljubljana, Ljubljana, Slovenia; 2 Department of Biotechnology, Jožef Stefan Institute, Ljubljana, Slovenia; University of Alberta, CANADA

## Abstract

Over the last 2 decades, several coronaviruses (CoVs) have crossed the species barrier into humans, causing highly prevalent and severe respiratory diseases, often with fatal outcomes. CoVs are a large group of enveloped, single-stranded, positive-sense RNA viruses, which encode large replicase polyproteins that are processed by viral peptidases to generate the nonstructural proteins (Nsps) that mediate viral RNA synthesis. Papain-like peptidases (PLPs) and chymotrypsin-like cysteine 3C-like peptidase are essential for coronaviral replication and represent attractive antiviral drug targets. Furthermore, CoVs utilize the activation of their envelope spike glycoproteins by host cell peptidases to gain entry into cells. CoVs have evolved multiple strategies for spike protein activation, including the utilization of lysosomal cysteine cathepsins. In this review, viral and host peptidases involved in CoV cell entry and replication are discussed in depth, with an emphasis on papain-like cysteine cathepsins. Furthermore, important findings on cysteine peptidase inhibitors with regard to virus attenuation are highlighted as well as the potential of such inhibitors for future treatment strategies for CoV-related diseases.

## Introduction

Coronaviruses (CoVs) belong to the Coronaviridae family within the Nidovirales order and are pleomorphic, enveloped, positive-strand RNA viruses with unusually large genomes, containing 27 to 32 kilobases. According to phylogenetic analyses, CoVs are classified into 4 genera, i.e., alpha, beta, gamma, and delta CoVs, which are further subdivided into several lineages. Alpha and beta CoVs are known to cause respiratory or intestinal infections in humans and other mammals, while gamma and delta CoVs predominantly affect birds [1, 2]. The first discovered CoV, known as infectious bronchitis virus, was isolated from chicken embryos already in 1937 [3]. Later, in the 1960s, the first 2 strains of human coronaviruses (HCoVs), HCoV-229E [4] and HCoV-OC43 [5], were discovered. Although they were endemic to the human population, accounting for 10% to 30% of common colds, they were not given much importance as they caused only mild symptoms, and more severe diseases developed only in elderly, newborn, and immunocompromised individuals [6–8]. However, this view changed at the beginning of the 21st century with the realization that CoVs are maintained within an animal reservoir from which their transmission to humans via intermediate host species is possible [9].

Such a spillover occurred in November 2002 in Foshan, Guangdong Province, China, where patients displayed symptoms of atypical pneumonia, which later became known as Severe Acute Respiratory Syndrome (SARS) [10]. A decade later, a novel CoV was identified in patients from the Arabian Peninsula in June of 2012 [11]. The clinical features of the underlying disease, called the Middle East Respiratory Syndrome (MERS), were similar to SARS, with additional gastrointestinal symptoms and frequent acute kidney failure. The differences might be explained by disparate preferences for the entry receptor [12], whereas dipeptidyl peptidase 4 (DPP4) is involved in Middle East Respiratory Syndrome Coronavirus (MERS-CoV) infection [13], and angiotensin-converting enzyme 2 (ACE2) is involved in Severe Acute Respiratory Syndrome Coronavirus (SARS-CoV) infection [14]. Both zoonotic viruses presumably originated in horseshoe bats and acquired adaptations to jump to humans from their intermediate hosts, including domestic Himalayan palm civet cats, raccoon dogs, Chinese ferret badgers (in the case of SARS-CoV), and dromedary camels (in the case of MERS-CoV) [1, 15, 16].

Since then, 2 additional strains of HCoV were found to be circulating in the human population, HCoV-NL63 [17] and HCoV-HKU1 [18], which result in milder symptoms and are, similarly to HCoV-229E and HCoV-OC43, well adapted to humans [19]. The emergence of another pandemic CoV zoonosis seemed very likely, as horseshoe bats were suggested to harbor viral variants capable of infecting human cells without the need for prior mutations [20]. Indeed, at the end of 2019, Wuhan, Hubei Province, China became the hotspot for the spread of a novel HCoV, initially termed as “novel coronavirus (2019-nCoV),” but subsequently renamed Severe Acute Respiratory Syndrome Coronavirus 2 (SARS-CoV-2) [21]. It has been shown that SARS-CoV-2 shares an 80% sequence similarity to its predecessor SARS-CoV and only 50% with MERS-CoV [22]. Nevertheless, SARS-CoV-2 reached practically all parts of the globe, causing one of the most severe pandemics, Coronavirus Disease 2019 (COVID-19), in recent history [23].

COVID-19 spreads via droplets and contact with infected surfaces [24, 25], and the fecal–oral route of transmission is also possible [26]. The main symptoms of COVID-19 include fever, weakness, dry cough, myalgia, and dyspnea. The severity of COVID-19 widely ranges from a mild, uncomplicated illness to bilateral pneumonia with acute respiratory distress syndrome, sepsis, and multi-organ failure, including kidney failure, cardiac injury, and thrombosis [27, 28]. The elderly population is more susceptible to disease, and a significant number of patients display comorbidities such as diabetes, hypertension, and cardiovascular disease [29].

Besides symptomatic treatment, several approved therapeutics have been tested for the antiviral treatment of COVID-19, and many more are undergoing preclinical development. To stop successful entry into the cell, as well as viral replication and propagation, the inactivation of host or viral peptidase activities or even both is necessary. Spike surface envelope glycoprotein, one of four structural proteins [30], is needed for virion entry into target cells, which is facilitated by binding to cell receptors (e.g., ACE2, DPP4, or aminopeptidase N) and fusion of viral and cell membranes. The large ectodomain of the spike protein is a target for several host peptidases, which cleave it at the border of domains S1 and S2 (cleavage site S1/S2) and at the S2’ site [31]. The mechanism of their action is highly pH dependent and involves different enzymes at various stages of the viral life cycle. Furthermore, cleavage takes place on the cell membrane, in endosomes, extracellularly, or during protein biosynthesis in the endoplasmic reticulum/Golgi apparatus. Differences in cleavage events may modulate viral pathogenicity and host range as well as cell and tissue tropism [31–33].

**Fig 1:**
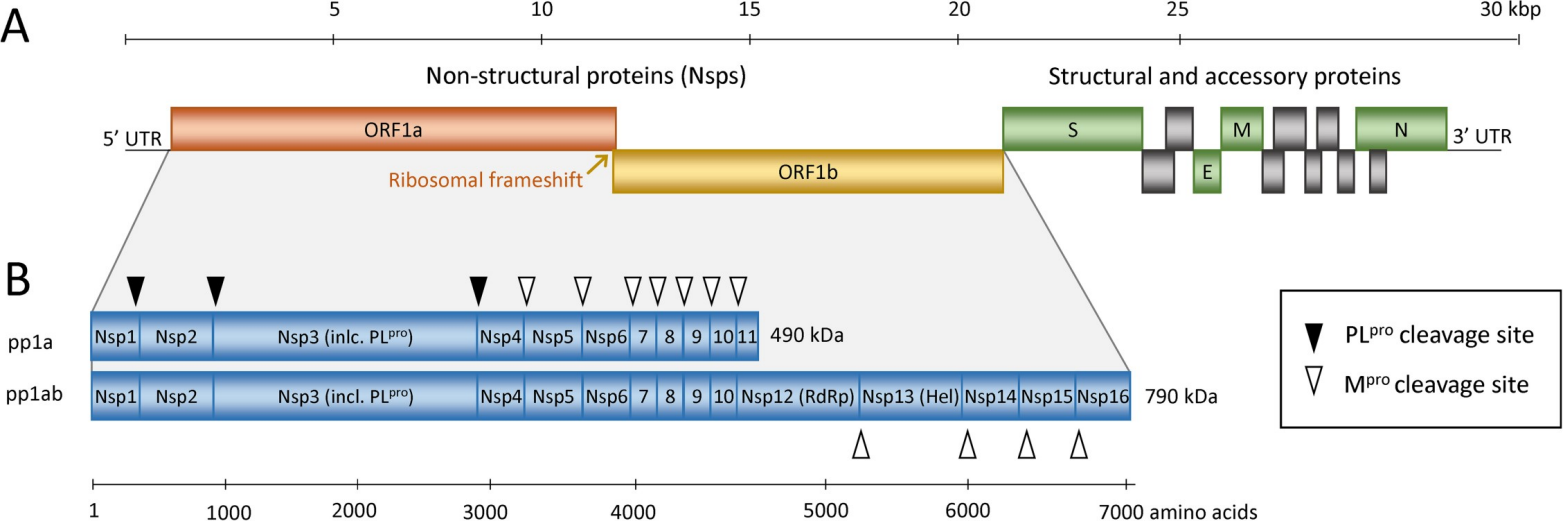
**Schematic representation of the SARS-CoV and SARS-CoV-2 genome (A) and the replicase polyproteins (B).** The genomic RNA comprises 2 parts, of which the first part (ORF1a and ORF1b) directly translates into 2 polyproteins pp1a and pp1ab due to a −1 frameshift between the 2 ORFs (orange arrow). They are composed of 16 Nsps (Nsp1–Nsp16) that form the replication–transcription complex. The polyproteins are processed into mature Nsps by the 2 peptidases as indicated in panel B by black and white triangles, namely M^pro^, which is Nsp5, cleaves the polyprotein at 11 sites and PL^pro^, which is part of the Nsp3, performs cleavage at 3 sites. Among the released Nsps are RdRp (Nsp12) and RNA helicase (Nsp13). The 3′ third of the genome encodes the structural and accessory proteins, including spike (S), envelope (E), membrane (M), and nucleocapsid (N) shown in green and gray. Nsp, nonstructural protein; ORF, open reading frame; RdRp, RNA-dependent RNA polymerase; SARS-CoV, Severe Acute Respiratory Syndrome Coronavirus; SARS-CoV-2, Severe Acute Respiratory Syndrome Coronavirus 2.

The viral genome encodes the cysteine papain-like peptidases (PLPs) and chymotrypsin-like cysteine 3C-like peptidase (3CL^Pro^), also known as main peptidase (M^Pro^) [34, 35], which are necessary for mediating viral genome transcription and replication. The viral RNA genome is translated into 2 large polyproteins, pp1a and pp1ab, which are, by their intrinsic peptidase activity, further cleaved into several nonstructural proteins (Nsps) as presented in Fig 1, building a replicase–transcriptase complex that is vital for viral transcription and replication [32, 33, 36, 53].

Many studies have recently been accelerated to provide key insights into the mechanisms underlying SARS-CoV-2 infection and to determine potential targets for antiviral interventions. Viral and host cysteine peptidases have become promising targets for antiviral treatment. In this review, we explore CoV-related promoting activities of viral and host peptidases with an emphasis on cysteine peptidases and discuss the possibilities of using their inhibitors to mitigate HCoV infections.

### Viral and host peptidases in CoV cell entry and replication

Peptidases play important roles in key steps of CoV infection and replication (reviewed in [31, 36]). Both viral and host peptidases are involved in these processes (Table 1). The peptidases encoded in the viral genome are essential for replicase polyprotein processing and evasion of the host immune response, while host peptidases are involved in different steps of virion uptake into the host cell.

**Table 1:** The peptidases involved in the infection and replication of CoVs.

Peptidase	Catalytic type	Function	Origin	CoV	Reference
Chymotrypsin-like peptidase 3CL^pro^ (M^pro^)	Cysteine	Polyprotein processing	Viral	All	Reviewed in [39]
PLP PL1^pro^	Cysteine	Polyprotein processing, immune system evasion	Viral	α-CoV (NL63, 229E), β-CoV (HKU1, OC43)	Reviewed in [51]
PLP PL2^pro^	Cysteine	Polyprotein processing, immune system evasion	Viral	All	Reviewed in [51]
ACE2	Metallo (zinc)	Receptor	Host	SARS, NL63	Reviewed in [15, 62]
Aminopeptidase N	Metallo (zinc)	Receptor	Host	229E	[67]
DPP4	Serine	Receptor	Host	MERS	[13]
TMPRSS2	Serine	Spike protein proteolytic activation	Host	SARS, MERS, 229E, OC43, HKU1, SARS-CoV-2	[61, 75, 76, 78–81]
Cathepsins L and B	Cysteine	Spike protein proteolytic activation	Host	SARS, MERS	[82, 86]

ACE2, angiotensin-converting enzyme 2; α-CoV, alphacoronavirus; β-CoV, betacoronavirus; CoV, coronavirus; DPP4, dipeptidyl peptidase 4; PLP, papain-like peptidase; MERS, Middle East Respiratory Syndrome; M^Pro^, main peptidase; SARS, Severe Acute Respiratory Syndrome; SARS-CoV-2, Severe Acute Respiratory Syndrome Coronavirus 2; TMPRSS2, transmembrane peptidase/serine subfamily member 2.

### CoV-encoded peptidases

The genomes of CoVs encode the PLPs, papain-like (PL)1^pro^ and PL2^pro^, within Nsp3 and 3CL^pro^ within Nsp5 (reviewed in [15, 37]). 3CL^pro^, also named M^pro^, is composed of 3 domains; domains 1 and 2 form the chymotrypsin-like fold with the substrate-binding site located in the cleft between the 2 domains, and domain 3 is required for dimer formation and affects catalytic activity by dynamically driven allostery. M^pro^ is active only as a dimer and processes the polyprotein at 11 distinct cleavage sites with 2 self-cleavage sites that are cut most efficiently. Both the substrate-binding and dimerization sites have been targeted for inhibitory drug development [38–45]. CoV M^pro^ shares highly conserved substrate recognition pockets, which are responsible for cleaving the viral polyprotein and host factors involved in the innate immune response, including the signal transducer and activator of transcription 2 and the signaling protein nuclear factor-κB essential modulator [15, 46–48]. Recently, conservation at the polyprotein cleavage sites has been confirmed. All 11 M^pro^ sites are highly conserved or identical, as only 12 out of the 306 residues of the SARS-CoV-2 M^pro^ sequence are different from those of SARS-CoV [49]. Additionally, a proteomic analysis of cellular responses indicated that SARS M^pro^ also affects cellular protein metabolism and modification, particularly in the ubiquitin proteasome pathway [50].

The other CoV peptidases, PLPs, are multifunctional enzymes that are involved in polyprotein processing but also play important roles in viral interactions with the host (reviewed in [37, 51]). They are primarily responsible for the cleavage of the N-terminal part of the polyprotein and catalyze replicase polyprotein processing at the cleavage sites between Nsp1/Nsp2, Nsp2/Nsp3, and Nsp3/Nsp4 [52]. CoVs encode either a single PLP (PL^pro^) or 2 PLPs (PL1^pro^ and PL2^pro^) that process a total of 3 cleavage sites within the polyprotein [51, 53]. SARS-CoV encodes only 1 PLP domain (PL^pro^) within Nsp3, which belongs to the peptidase clan CA in the MEROPS peptidase classification. The active site contains a classic catalytic triad, composed of Cys112-His273-Asp287, and catalyzes viral polyprotein processing at the N-terminus of pp1a, releasing the Nsps Nsp1, Nsp2, and Nsp3 through recognition of a LeuXaaGlyGly (LXGG) motif [36, 54]. Beyond cleaving viral polyproteins, PLPs exert additional activities that promote virus replication. These enzymes resemble the structure of human ubiquitin-specific peptidases and are thereby known as viral ubiquitin-specific peptidases, often acting as deubiquitinating enzymes with the ability to remove the posttranslational modification of ubiquitin from target proteins [55]. The *in vitro* characterization of PL^pro^ enzymatic activities reveals that PL^pro^ can recognize and hydrolyze the cellular proteins ubiquitin and ubiquitin-like protein interferon-stimulated gene product 15 (ISG15), both bearing the LXGG recognition motif at their carboxyl terminus ([56]; reviewed in [36]). The dual deubiquitinase (ubiquitin cleaving) and deISGylase (ISG cleaving) activities of SARS-CoV PL^pro^ antagonize the type I interferon (IFN-I) response and were suggested to be a key viral immune response mechanism [57–59]. Additionally, SARS-CoV PL^pro^ has been shown to inhibit interferon regulatory factor 3 phosphorylation early in the IFN-I signaling pathway [57, 58]. SARS-CoV PL^pro^ is able to inhibit interferon regulatory factor 3-dependent IFN-I activation at later stages as well, by deubiquitinating interferon regulatory factor 3 and blocking the ability to induce IFN-β transcription, suggesting innate immune antagonistic activities of PL^pro^ that may contribute to SARS-CoV pathogenesis [60].

M^pro^ and PL^pro^ catalyze their own release and liberate other Nsps from the polyprotein. As such, the enzymatic activities of both viral peptidases are essential for the viral life cycle. Nevertheless, host cell peptidases are also required for viral infectivity (reviewed in [30]).

### Host peptidases are essential for virion uptake

Host peptidases are critical for CoV entry and act either as receptors for the attachment of the virion spike protein to the target cell or as facilitators of virion envelope fusion with the target cell membrane. The metallo-carboxypeptidase ACE2 is the primary receptor for the SARS-CoV, HCoV-NL63, and novel SARS-CoV-2 [15, 61, 62]. The peptidase activity of ACE2 negatively regulates the renin–angiotensin system, which plays a key role in maintaining blood pressure homeostasis as well as fluid and salt balance in mammals (reviewed in [63]). Additionally, the unique binding of SARS-CoV spike protein to ACE2 induces ACE2 ectodomain shedding, which leads to the down-regulation of ACE2. Since normal ACE2 expression protects from lung injury, reduced ACE2 levels are believed to promote lung pathogenesis. ACE2 ectodomain shedding is mediated by the metallopeptidase ADAM17, a member of the a disintegrin and metalloproteinase (ADAM) family of peptidases, also named tumor necrosis factor alpha (TNF-α)-converting enzyme [64–66]. The metallopeptidase aminopeptidase N (CD13) is the receptor for HCoV-229E, which utilizes the endosomal pathway for cell entry, in which lysosomal peptidases, including cathepsin L, are involved [67]. DPP4 (CD26) is a serine aminopeptidase that acts as a receptor for MERS-CoV and is also an essential determinant of MERS-CoV tropism [13, 68]. Following binding to the host cell receptor, additional factors affect the next steps of virion entry. For example, the transmembrane proteins tetraspanins (e.g., tetraspanin CD9) facilitate MERS-CoV entry by forming complexes between DPP4 and the type II transmembrane serine peptidases, which are important fusion-activating peptidases that facilitate virion entry and promote efficient infection [69]. After the spike protein binds to the cellular receptor, it is subjected to limited proteolysis at distinct cleavage sites located at the domain boundary (S1/S2) or within domain 2 upstream of the putative fusion peptide (S2’), which directly leads to membrane fusion.

Peptidases involved in CoV spike protein activation can act at different stages of the viral life cycle and include proprotein convertases (e.g., furin) during virus packaging, extracellular peptidases (e.g., elastase), cell surface peptidases (e.g., transmembrane serine peptidases) after receptor binding, and lysosomal peptidases (e.g., cathepsins L and B) after virion endocytosis (reviewed in [31, 70]). Proprotein convertase furin, a subtilisin-like serine peptidase, is not involved in activating MERS for virion entry but rather in determining cell tropism [71, 72]. Furthermore, a novel furin cleavage site at the S1/S2 boundary has been identified with a bioinformatics analysis of the novel SARS-CoV-2, indicating possible effects of this virus on cell tropism [73]. The extracellular serine peptidase elastase, which is secreted by neutrophils as part of an inflammatory response to viral infection, was shown to enhance SARS-CoV infection by proteolytic activation of the SARS-CoV spike protein [74]. The SARS-CoV spike protein can be proteolytically activated by a human airway and alveolar peptidase transmembrane peptidase/serine subfamily member 2 (TMPRSS2) as well as other transmembrane serine peptidases, including human airway trypsin-like peptidase [75–77]. Furthermore, TMPRSS2 also activates spike protein for virion entry of MERS-CoV [78, 79] and other HCoVs, including HCoV-229E [80], HCoV-OC43, HCoV-HKU1 [81], and SARS-CoV-2 [61]. In addition to the TMPRSS2-facilitated direct entry from the cell membrane, the CoV also utilizes the endosomal pathway, where spike proteins are proteolytically activated by the lysosomal cysteine peptidases cathepsin L or cathepsin B. Among HCoVs, this is the case for the SARS-CoV [82] and MERS-CoV [86]. Between the 2 distinct virion entry pathways, namely the endosomal pathway and the direct cell surface entry, clinical isolates of HCoV-229E, HCoV-OC43, and HCoV-HKU1 have demonstrated that direct entry from the cell surface or early endosomes is preferred *in vivo* [80, 81]. Furthermore, factor Xa [83] and an unidentified leupeptin-sensitive peptidase [84] are other examples of peptidases that were shown to activate SARS-CoV spike protein for entry into host cells. Another cysteine peptidase, m-calpain, has been implicated in SARS-CoV replication, indicating an important role for m-calpain during the early steps of the SARS-CoV life cycle [85]. Taken together, host cell peptidases are indeed important for virion uptake into the host cell (Table 1), and host cysteine peptidases have been recognized as key players in virion entry and will be addressed in more detail in the following section.

### Host papain-like cysteine cathepsins are involved in CoV uptake

**Fig 2:**
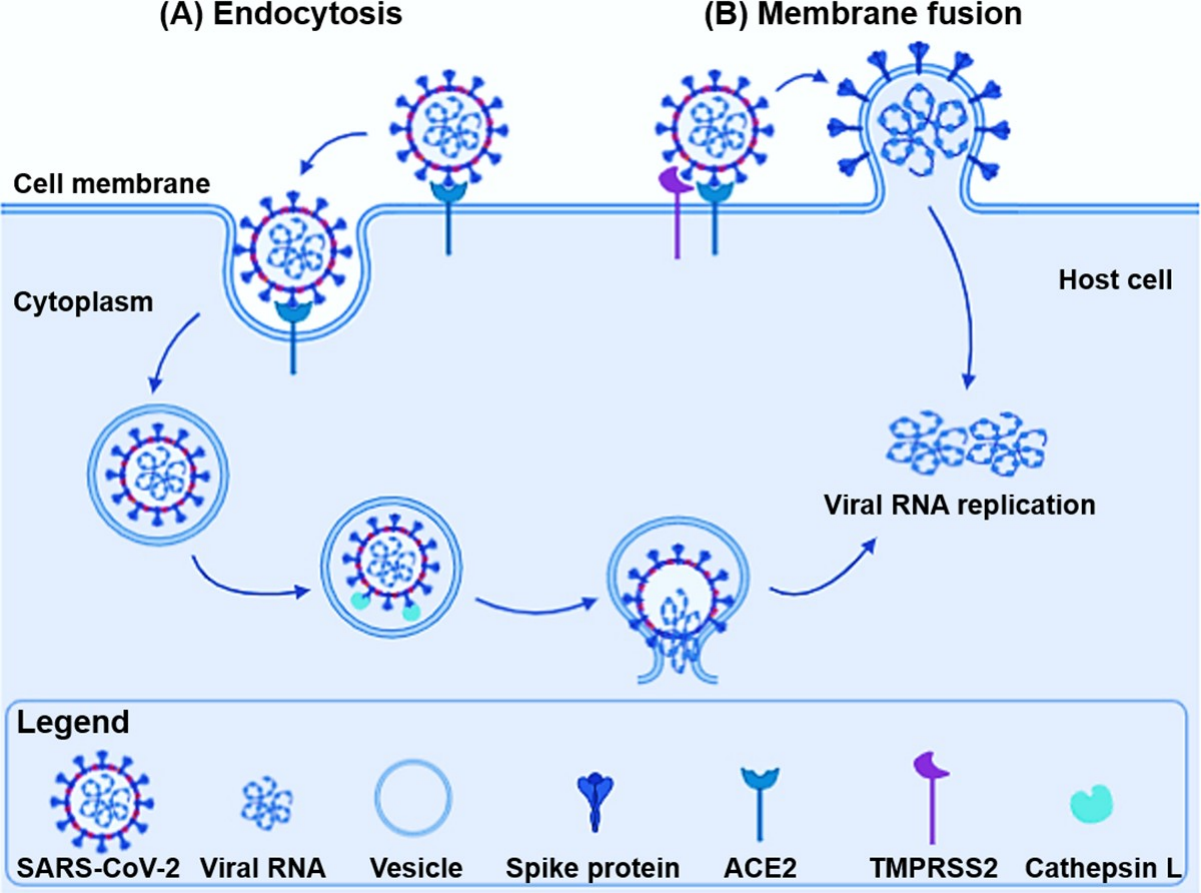
The routes of SARS-CoV entry into target cells. The viral entry takes place either by (A) receptor-mediated endocytosis or by (B) membrane fusion. (A) Binding of SARS-CoV and SARS-CoV-2 to the cellular receptor ACE2 can result in virion uptake into endosomes. The spike protein is activated by cysteine peptidase cathepsin L in endo-lysosomes. (B) In membrane fusion, the spike protein activation is mediated by TMPRSS2 or other transmembrane serine peptidases at the cell surface, resulting in fusion of the viral membrane with the plasma membrane. Either way, the RNA genetic material of the virus is released, and the late stage of the life cycle subsequently takes place by RNA replication. ACE2, angiotensin-converting enzyme 2; SARS-CoV, Severe Acute Respiratory Syndrome Coronavirus; SARS-CoV-2, Severe Acute Respiratory Syndrome Coronavirus 2; TMPRSS2, transmembrane peptidase/serine subfamily member 2.

CoVs, including SARS-CoVs, can employ 2 routes for host cell entry that are determined by the localization of the peptidases required for spike protein activation (Fig 2). Spike proteins can be activated by transmembrane serine peptidases at the cell membrane, resulting in fusion of the viral membrane with the plasma membrane [30]. Alternatively, a virion gains entry into a target cell by binding to the receptors on the cell surface (e.g., ACE2), resulting in its uptake into endosomes [87]. Endosomal vesicles within the target cells form highly dynamic multifunctional cellular compartments that contain multiple proteolytic enzymes, including lysosomal peptidases [88]. The main class of lysosomal peptidases comprises the cathepsins, which are subdivided into 3 subgroups according to the active site amino acid residue: aspartyl (cathepsins D and E), serine (cathepsins A and G), and cysteine cathepsins. The latter constitute the largest cathepsin family, with 11 peptidases in humans belonging to clan CA, family C1a: cathepsins B, C/DPP1, F, H, K, L, O, S, W, V, and Z/X [89]. They are delivered to early endosomes as inactive zymogens and are activated by the lower pH either through proteolytic processing by other endosomal/lysosomal hydrolases or through their interactions with glycosaminoglycans [90–92]. Cysteine cathepsins are optimally active and stable at acidic pH and, generally, inactivated at neutral pH; although some of them, e.g., cathepsin S, maintain their proteolytic activity at neutral pH [91, 93]. Cathepsins were initially considered intracellular enzymes, responsible for the nonspecific bulk proteolysis in the acidic environment of the endosomal/lysosomal compartments [93, 94]. Later, a broad spectrum of more specific roles was proposed for cathepsins, under both physiological and pathological conditions [94–99], including virus infections [30].

The role of cysteine cathepsins in the viral life cycle is particularly associated with virion entry. In non-enveloped viruses (i.e., reoviruses), the virion is converted into an infectious subvirion particle via partial proteolysis, mediated by cathepsins B, L, or S [171–175]. In more detail, Ebert and colleagues showed that cathepsin L or cathepsin B is required for reovirus entry into murine fibroblasts L929 [172]. Two years later, Golden and colleagues showed that acid-independent cathepsin S mediated outer capsid processing in macrophage-like P388D cells supported infection by virions of strain Lang, but not strain c43 [175]. In the last decade, the proteolytic digestion by endosomal cathepsins has been described during entry of enveloped viruses, such as the Ebola virus and CoVs [30, 176]. Cathepsin L is most commonly associated with the activation of viral glycoproteins. It is known to process the SARS-CoV spike protein as well as many proteins of other HCoVs, including MERS-CoV and HCoV-229E [67, 79, 101]. The cysteine peptidase cathepsin B can also be involved in virion entry and exhibits distinct properties compared to cathepsin L and other cathepsins; it generally has a higher pH optimum and, while preferring an aromatic P2 residue, can process dibasic substrates [177, 178]. Cathepsin B was shown to play key roles in the lifecycle of the Ebola virus [176], Nipah virus [179], and feline CoV [180], catalytically activating viral membrane glycoproteins, which leads to fusion of the viral envelope with the endosomal membrane and virion release from endosomes into the cytoplasm [181, 182].

Interestingly, it has been shown that the SARS-CoV, but not HCoV-NL63, utilizes cathepsins to enter the host cell [100]. Furthermore, only cathepsin L, but not other cathepsins, primes the SARS-CoV for membrane fusion, in which the interaction with ACE2 is required [101, 102]. Based on these observations, a 3-step process of SARS-CoV and SARS-CoV-2 infection has been proposed (Fig 2): receptor binding, induced conformational changes in spike protein, and cathepsin L proteolysis within endosomes [101]. As mentioned above, the spike protein in the SARS-CoV exposes a peptidase-accessible loop between the S1 and S2 subunits, which can be targeted by different enzymes, including TMPRSS2, furin, and cathepsin L, thus activating the membrane fusion function of the spike protein. The SARS-CoV can use the endosomal cathepsins B or L for spike protein priming in TMPRSS2^-^ cells [101]. However, spike protein priming by TMPRSS2, but not by cathepsins B and L, was proposed to be essential for virion entry into the primary target cells and for viral spread in the infected host [103–105]. Nevertheless, a recent study [61] has demonstrated that SARS-CoV-2 infection was inhibited by a serine peptidase inhibitor, likely reflecting spike protein priming by other peptidases. Camostat mesylate, which is active against TMPRSS2 [104], only partially blocked SARS-CoV-2 spike protein-driven entry into Caco-2 cells and Vero-TMPRSS2^+^ cells, whereas full inhibition was attained in combination with E-64d, a broad inhibitor for cathepsins B, H, L, and calpain [106]. This suggests that SARS-CoV-2 spike protein can use both cathepsins B and L as well as TMPRSS2 for priming in these cells. Another study confirmed the important role of cysteine cathepsins in SARS-CoV-2 entry and identified cathepsin L as a critical lysosomal peptidase for virion entry into human embryonic kidney 293 cells stably expressing recombinant human ACE2, while the cathepsin B–specific inhibitor CA-074 [107] did not exhibit any marked effect on SARS-CoV-2 entry [77].

### The impact of cysteine peptidases on the CoV host immune response

Studies on the effect of SARS-CoV-2 on the host immune response are limited; however, preliminary data show great similarity with other beta CoVs with similar genomic sequences [22, 108]. The antiviral immune response starts with the recognition of the virus and virally infected cells by the innate immune system. Viral RNA is recognized by pathogen-associated molecular pattern receptors, such as Toll-like receptors (TLRs) [109]. The activation of nucleic acid–sensing TLRs (i.e., TLR-3, TLR-7, and TLR-9) is a complex process and is thought to involve a combination of asparagine endopeptidase (AEP) legumain and/or multiple cathepsins [110–113]. TLR trigger results in IFN-I secretion, which controls viral replication and supports the effective adaptive immune response [109]. Nevertheless, in senescent BALB/c mice administered with SARS-CoV intranasally, it was shown that SARS-CoV infection is able to delay the production of IFN-I, while the virus replicates rapidly, which results in diminished responses and numbers of immune effector cells [114, 115]. SARS-CoV-2 may develop even higher replication rates and lower induction of host IFN-I compared to those of SARS-CoV [116]. A recent clinical study showed that severely ill COVID-19 patients exhibited an impaired type IFN-I response characterized by low IFN production and activity, with subsequent down-regulation of IFN-stimulated genes [117].

To activate adaptive immunity, viral antigens must be presented to T cell receptors by infected cells and other antigen-presenting cells via the major histocompatibility complex class I or class II molecules. Together with other lysosomal peptidases and immunoproteasomes, cysteine cathepsins enable the formation of antigen epitopes or, by cleaving the invariant chain Ii, the priming of major histocompatibility complex II molecules and their trafficking to the cell surface [118–120]. SARS-CoV-infected monocytes down-regulate the expression of cathepsins involved in antigen presentation and processing, suggesting a limited activation of a favorable adaptive immune response against SARS-CoV [121].

An increasing number of studies are reporting lymphopenia as one of the hallmarks of severe COVID-19, which is exacerbated with disease progression [117, 122–125]. The most affected lymphocyte subsets are natural killer (NK) cells [126], cytotoxic CD8^+^ T lymphocytes (CTLs) [126, 127], and most CD4^+^ lymphocytes [127]. So far, several potential mechanisms that lead to lymphocyte deficiency in COVID-19 disease have been proposed, such as induced lymphocyte apoptosis, ACE2 receptor expression, or direct viral targeting of lymphoid organs [123]. In addition to apoptosis, inflammatory cell death (e.g., necroptosis or pyroptosis) cannot be excluded, as virulent HCoVs have been shown to promote RIPK3-dependent necroptosis and caspase-1-dependent pyroptosis [128]. Furthermore, functional assessments of NK cells and CTLs in COVID-19 patients showed that both their numbers and functions are compromised. NK cells and CTLs from COVID-19 patients exhibit increased expression of inhibitory receptor NKG2A and decreased expression of CD170a and effector molecules such as interferon gamma (IFNγ), TNF-α, and granzyme B, suggesting a state of exhaustion [126]. Therefore, virus-induced cytopathic effects that lead to immune insufficiency and dysregulation of immune response enable viral replication and cause tissue damage that is a major cause of COVID-19 pulmonary pathology. This has been confirmed by histologically examining lung tissue of severe cases of SARS-CoV-2 infection, which revealed infiltrates of inflammatory mononuclear cells and cytopathic-like changes [127].

In COVID-19 patients, transcriptomic analysis of peripheral blood mononuclear cells showed a significant increase in the expression of cathepsin L and B genes associated with the apoptotic pathway [129]. Cathepsins initiate apoptosis by cleavage of the proapoptotic molecule BH3-interacting domain death agonist (BID) and degradation of antiapoptotic Bcl-2 proteins. This results in the release of cytochrome *c* from mitochondria and the activation of caspases 3 and 7 [130, 131]. Furthermore, inhibitor studies suggest that cathepsins, in particular cathepsins B, C, and D, are key peptidases involved in inflammatory cell death [132]. The cytotoxic function of NK cells and CTLs is regulated by cathepsins. Perforin and granzymes are stored in their precursor forms in the cytotoxic granules of NK cells and CTLs. Upon cell activation, cathepsins C and H catalyze the conversion of progranzymes into granzymes and cathepsin L, although not redundantly, activates perforin. The function of these cathepsins is further regulated by the endogenous inhibitor cystatin F [133]; however, the status of cystatin F and cathepsins in cytotoxic cells in COVID-19 patients has not been evaluated so far.

### Virus attenuation by cysteine peptidase inhibitors

Targeting peptidases involved in the infection and replication of CoVs represents an effective strategy to block viral replication. An attractive target for development of antiviral drugs directed against the SARS-CoV-2 and other CoVs is 3CL^pro^ due to its essential role in processing polyproteins after translation. After the 2003 SARS-CoV outbreak, numerous inhibitors of 3CL^pro^ peptidase were proposed, yet no new drug candidates have succeeded in entering clinical trials (reviewed in [34, 134, 135]). However, several peptidic and small molecular inhibitors have exhibited potent activities against SARS-CoV 3CL^pro^ as reviewed in detail in the recent papers by others [136, 137]. Among other keto peptidomimetics, α-ketoamide inhibitors have been developed as potent inhibitors [45]. Similarly, the PLP domain of SARS-CoV Nsp3 has emerged as a viable drug target, resulting in the development of several SARS-CoV PL^pro^ inhibitors (reviewed by [36]). Recently, naphthalene-based PL^pro^ inhibitors were shown to be effective at halting SARS-CoV-2 PL^pro^ activity as well as SARS-CoV-2 replication, which might represent a strategy for generating PL^pro^-targeted therapeutics to be used against SARS-CoV-2 [138]. Next, peptides derived from HKU1 CoV were shown as effective inhibitors of formyl peptide receptor that plays important role in recognition by phagocytic cells [138].

In addition to viral peptidases, host peptidases are important for virus infection. As mentioned above, CoVs, including SARS-CoV and novel SARS-CoV-2, utilize a cathepsin-dependent endosomal pathway in addition to the direct cell surface serine peptidase-mediated pathway [30, 61, 77]. This suggests that targeting cysteine cathepsins could be a promising strategy for the development of antiviral drugs directed against SARS-CoVs as well as other related CoV infections.

The potent effect of cysteine peptidase inhibitors on CoV replication was already shown in the 1990s (Table 2) when Colling and Grubb demonstrated the inhibition of CoV replication by cystatins C and D [139, 140]. Cystatins are general inhibitors of cysteine peptidases (reviewed in [141–143]) and may inactivate either viral or host cysteine peptidases. Cystatin C potently affects HCoV-OC43 and HCoV-229E, inhibiting their replication by more than 99% at a concentration of 0.1 mM [140]. Cystatin D is specifically expressed in human saliva and tears [144] and inhibits CoV replication at physiological concentrations [139].

**Table 2:** A summary of cysteine cathepsin inhibitors tested for CoV inhibition.

Inhibitor	Peptidase(s)	Type	CoV	Target	Reference
Cystatin C	Cysteine peptidases	Protein	HCoV OC43 HCoV 229E	Virus replication	[140]
Cystatin D	Cysteine peptidases	Protein	HCoV OC43 HCoV 229E	Virus replication	[139]
E-64d (EST)	Cysteine peptidases	Epoxysuccinyl	SARS-CoV SARS-CoV-2	Virion entry	[77] [104]
K11777	Cysteine peptidases	Vinylsulfone	SARS-CoV	Virion entry	[105]
CID 23631927	Cathepsin L	Tetrahydroquinoline oxocarbazate derivative	SARS-CoV	Virion entry	[145]
SID 26681509	Cathepsin L	Thiocarbazate	SARS-CoV-2	Virion entry	[77]
Amantadine	Cathepsin L	Amantadine	SARS-CoV-2	Cleavage of CoV spike protein, down-regulation of cathepsin L gene expression, virus replication	[146]
Teiocoplanin, dalbavancin, oritavancin	Cathepsin L	Glycopeptide antibiotics	MERS-CoV, SARS-CoV SARS-CoV-2	Virion entry, cleavage of CoV spike protein	[147, 148, 183]
SSAA09E1	Cathepsin L	Benzamide	MERS-CoV, SARS-CoV	Virion entry, cleavage of SARS-CoV spike protein	[82]
5705213 7402683	Cathepsin L	Triazin-N-cyanoglycinate	SARS-CoV	Virion entry, cleavage of SARS-CoV S protein	[149]
Dec-RVKR-CMK	Cathepsin L, cathepsin B, TMPRSS2, furin, trypsin, papain	Methylketone	MERS-CoV	Virion entry	[72]
MDL-28170	Cathepsin L calpain	Ketone	HCoV-229E, SARS-CoV	Virion entry	[101, 150]
Cathepsin L III inhibitor (**Z-FY(t-Bu)-DMK)**	Cathepsin L	Ketone	SARS-CoV	Virion entry	[104, 151]

CoV, coronavirus; HCoV, human coronavirus; MERS-CoV, Middle East Respiratory Syndrome Coronavirus; SARS-CoV, Severe Acute Respiratory Syndrome Coronavirus; SARS-CoV-2, Severe Acute Respiratory Syndrome Coronavirus 2.

**Fig 3:**
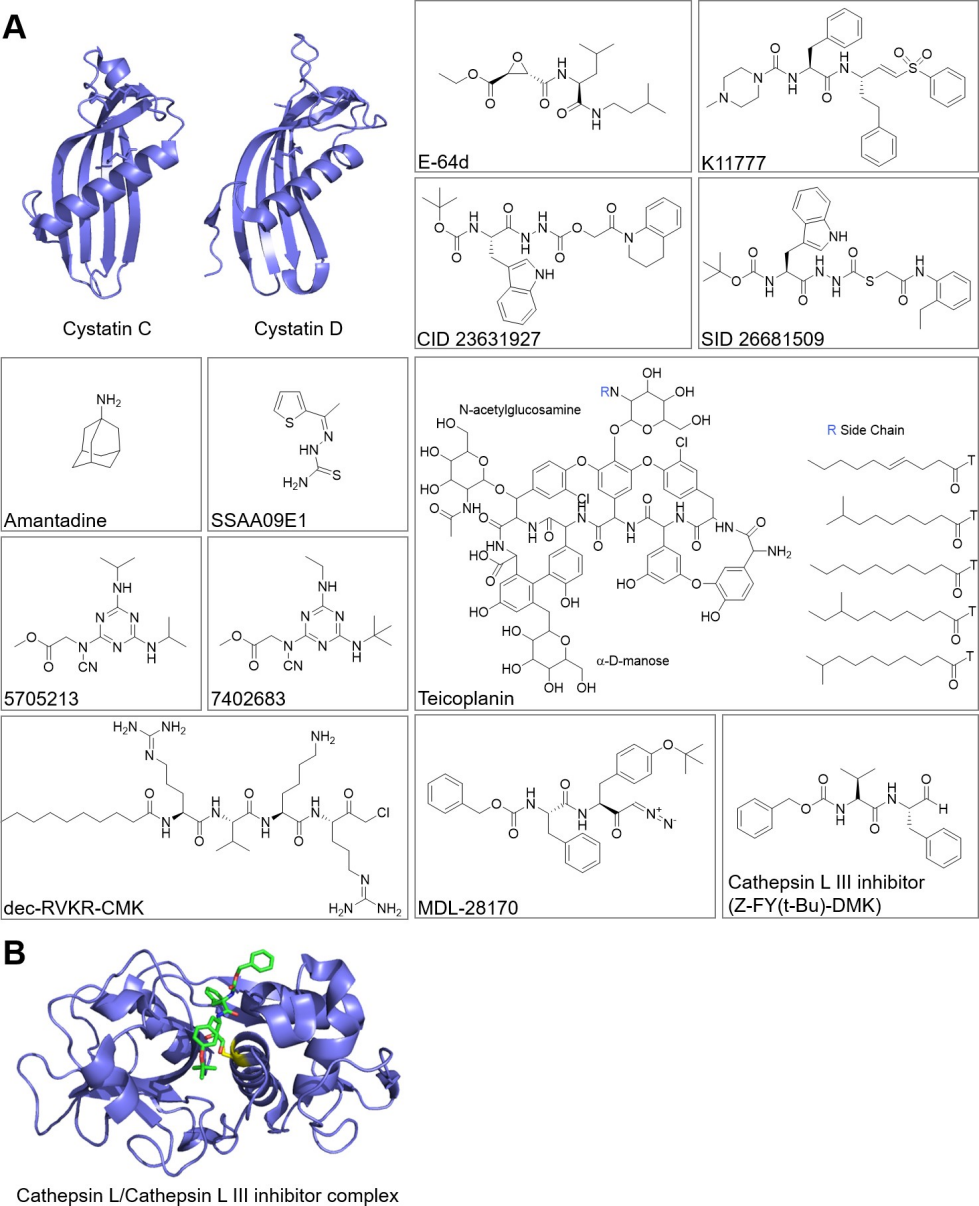
Structures of cysteine cathepsin inhibitors tested for CoV inhibition. (A) The structures of cysteine cathepsin inhibitors. PDB code for cystatin C is 3GAX and for cystatin D is 1RN7. (B) The crystal structure of cathepsin L in complex with cathepsin L III inhibitor (PDB code 3OF8). Cathepsin L is shown in blue, cathepsin L III inhibitor is represented by sticks and colored green, and cathepsin L active site cysteine is marked yellow. The structures were prepared using ChemDraw (PerkinElmer, United States of America), and crystal structures were prepared using PyMol (Schrödinger, USA). CoV, coronavirus; PDB, Protein Data Bank.

Similarly, the irreversible epoxysuccinyl cell permeable inhibitor E-64d (Fig 3) also provided an effective strategy to block virion entry into cells, as it blocked SARS-CoV entry into Caco-2 and Vero-TMPRSS2^+^ cells as well as SARS-CoV-2 entry into human embryonic kidney 293/hACE2 cells. For the latter, virion uptake was blocked by E-64d by 92.5% [61, 77]. Furthermore, simultaneous treatment with the cysteine peptidase inhibitor E-64d and serine peptidase inhibitor camostat strongly inhibited virion entry and multistep growth of the SARS-CoV in airway epithelial Calu-3 cells and TMPRSS2-expressing HeLa cells [104]. The cysteine peptidase inhibitors K11777 and closely related vinylsulfones also target cathepsin-mediated virion entry into cells and therefore act as broad-spectrum antiviral agents [105]. In particular, K11777 is very potent, inhibiting SARS-CoV as well as Ebola virion cell entry at subnanomolar concentrations.

Among the specific cysteine peptidase inhibitors, the targeting of cathepsin L has provided the most promising antiviral effects. The cathepsin L–specific inhibitor CID 23631927 was shown to block SARS-CoV entry in human embryonic kidney 293T cells [145]. Additionally, the activity-based probe, which labels active cathepsin L, confirmed the effect of the inhibitor on SARS-CoV entry into human cells [145]. Similarly, the cathepsin L–specific inhibitor SID 26681509 blocked SARS-CoV-2 entry into human embryonic kidney 293/hACE2 cells. In the same study, the cathepsin B–specific inhibitor CA-074 did not exert any marked effect on virion entry [77].

Another example of a drug that could impair the function of cathepsins involved in the cleavage of CoV spike protein is amantadine. As a prophylactic agent for influenza and Parkinson’s disease, it was shown to down-regulate cathepsin L expression at a concentration of 10 μM [146]. Additionally, amantadine acts as a lysosomotropic agent and disrupts the lysosomal pathway, interfering with the capacity of the virus to replicate [146]. Moreover, glycopeptide antibiotics such as teicoplanin and its derivatives dalbavancin, oritavancin, and telavacin underwent high-throughput screening of Food and Drug Administration (FDA)-approved drugs and were identified to prevent MERS-CoV, SARS-CoV, and Ebola pseudovirus cell entry due to cathepsin L inhibition [147, 148]. Teicoplanin was recently shown to prevent the entrance of SARS-CoV-2 pseudoviruses into the cytoplasm in low molecular concentration [183]. By screening a chemical library of compounds for blocking the entry of HIV-1 pseudotyped with SARS-CoV spike protein but not that of HIV-1 pseudotyped with vesicular stomatitis virus surface glycoprotein G, benzamide inhibitor SSAA09E1 was discovered as effective, again by inhibiting cathepsin L [82]. Small molecule inhibitors of cathepsin L that can impair virion entry and SARS-CoV spike protein cleavage were also identified in a high-throughput screening assay of peptides derived from the glycoproteins of the SARS-CoV and Ebola, Hendra, and Nipah viruses [149]. Among them, 5705213 and its derivative 7402683 inhibited cathepsin L-mediated cleavage of the recombinant SARS-CoV spike protein in a dose-dependent manner. Additionally, 5705213 in combination with the TMPRSS2 peptidase inhibitor completely blocked the entry of the pseudotypes bearing the SARS-CoV spike protein [149].

The entry of MERS-CoV into cells was also blocked by the furin inhibitor dec-RVKR-CMK (Decanoyl-Arg-Val-Lys-Arg-CMK) due to its inhibition of cathepsin L and TMPRSS2 enzymatic activity. In addition, dec-RVKR-CMK also inhibited cathepsin B, trypsin, and papain [72]. Another cathepsin L inhibitor that affects virus entry is MDL-28170 [101, 150]. This inhibitor blocked SARS-CoV infection of TMPRS2^-^ HeLa-ACE2 cells driven by spike protein. Additionally, pseudotype SARS-CoV infection of TMPRS2^-^ HeLa-ACE2 cells was inhibited by cathepsin L III inhibitor for 80% [104].

Together, various studies have identified an important role of cysteine cathepsins in virion entry (Table 2) and suggest that inhibition of cathepsins, especially cathepsin L, could be an effective strategy for the search/development of new drugs for the treatment of COVID-19. Although the role of cathepsin L seems pivotal, the role of other host cysteine peptidases in virus infection and replication should be further investigated to assess their individual contribution to virus spread.

### The potential of novel selective cysteine peptidase inhibitors

Exogenous cysteine peptidase inhibitors can be of protein or nonprotein origin and isolated from animals, microorganisms, plants, fungi, neutralizing monoclonal antibodies and their fragments, propeptides, or synthetic small molecules. Based on the mechanism of inhibition, we distinguish between reversible and irreversible exogenous inhibitors [152]. The majority of cysteine peptidase inhibitors contain a reactive electrophilic functional group that reacts with cysteine’s thiol group in the active site of the enzyme, where the peptide fragment is crucial for selectivity and enzyme recognition (reviewed in [90, 152–154]).

Reversible cysteine peptidase inhibitors can be peptide molecules such as propeptides, monoclonal antibodies, and small molecules, including aldehydes, methylketones, trifluoromethylketones, diketones, α-ketoacids, α-ketoesters, α-ketoamides, α-keto-β-aldehydes, nitriles, azapeptide nitriles, thiosemicarbazones, and biflavones (reviewed in [152–155]). Numerous promising reversible cathepsin L inhibitors exist, including aldehyde derivative iCP (Napsul-Ile-Trp-CHO) [156, 157], thiosemicarbazone derivative KGP94 [158], tetrahydroquinoline oxocarbazate derivative CID 23631927, and SID 26681509, which block SARS-CoV entry into human cells [77, 145, 159, 160]. Certain compounds that are already used in clinical practice are candidates for repurposing, of which the reversible cysteine peptidase inhibitor nitroxoline shows promise [160, 161]. This well-established antimicrobial agent used for the treatment of urinary tract infections was identified as a potent cathepsin B inhibitor both *in vitro* and *in vivo* in various cell-based functional assays and tumor mice models [160–162]. The antituberculosis agent rifampicin was also shown to inhibit cathepsins B, L, and H with inhibition constants in the low micromolar range [163]. In addition to classic small molecule inhibitors, organometallic complexes of quinolone, clioquinol, and nitroxoline with ruthenium have been identified as inhibitors of cysteine peptidases [162, 164].

The most studied irreversible inhibitors are epoxysuccinyl inhibitors, whose reactive electrophilic epoxide rings form covalent thioester bonds with the thiol group of the reactive active site. 1-[L-N-(trans-epoxysuccinyl)leucyl] amino-4-guanidinobutane (E-64) is the best known general epoxysuccinyl cysteine peptidase inhibitor, which was first isolated from *Aspergillus japonicus* [165]. Due to their high reactivity and lack of selectivity, E-64 and its cell-permeable derivative E-64d are not applicable to clinical practice. However, E-64 represents the lead compound for the development of new inhibitors with increased selectivity (reviewed in [90, 152, 153, 155]). As a derivative of E-64, the potent and selective cathepsin B inhibitor CA-074 was synthesized [107], and its low membrane permeability was improved by methylation of the carboxyl group [166]. CA-074Me is cell permeable and can be hydrolyzed to CA-074 within cells; however, such methylation decreases its selectivity for cathepsin B [166]. Based on the structure of E-64, the cathepsin L–specific inhibitor CLIK-148 was developed [167]. In addition to epoxysuccinyl inhibitors, irreversible cysteine peptidase inhibitors include epoxides, vinyl sulphones, aziridines, azomethylketones, halomethylketones, acyloxymethylketones, azapeptides, thiadiazoles, hydroxametes, β-lactams, and α,β-unsaturated carbonyl derivatives and others (reviewed in [152, 154]).

However, irreversible inhibitors are less suitable for use in clinical practice due to the presence of reactive electrophilic warheads, which can, in addition to targeting enzymes, nonspecifically react with nucleophiles from other off-target proteins, resulting in side effects. Furthermore, their bioavailability can be lower than that of reversible inhibitors due to their peptide scaffold (reviewed by [152–154]). Therefore, new research is directed toward the development of selective reversible peptidase inhibitors, while selective irreversible inhibitors remain important research tools in elucidating the molecular mechanisms of cathepsins in disease [90]. However, as host peptidases are also involved in normal physiological process, the care must be taken to avoid side effects that may occur. These are expected to be similar as those seen in treatment of other diseases where peptidase inhibitors are already used in clinical practice. In particular, the impact of new treatment on host immune system has to be considered [90, 168].

## Conclusions and future perspectives

CoVs have been recognized for more than 5 decades and represent a group of severely pathogenic viruses with a high risk to public health. Recurrent viral threats continue to emerge, most recently by the novel SARS-CoV-2, which developed into a pandemic of COVID-19 in 2020. So far, no approved vaccines or specific therapeutics exist for these pathogenic viruses, including SARS-CoV-2. Improved clinical outcomes of COVID-19 patients have been reported after treatment with the repurposed drug remdesivir, which inhibits viral RNA polymerases [169]. However, new antiviral drugs directed against the SARS-CoV-2 and other CoVs are urgently needed to provide adequate responses to future pandemic threats. Targeting viral and host peptidases involved in virion entry and replication could be a key for the development of new antiviral therapies. Peptidase inhibitors are already successfully used in clinical practice for treatment of other infectious diseases caused by viruses such as HIV, hepatitis, rhinovirus and other (reviewed in [170]). Nevertheless, a better understanding of the mechanisms by which host cysteine peptidases contribute to SARS-CoV-2 uptake into target cells would provide valuable new information for virus pathogenesis, vaccine design, and drug targeting. Peptidase inhibitors with dual inhibitory activity targeting both viral and host PLPs with an additional positive effect on the immune response may represent the needed multilayered quality of an effective anti-coronaviral therapeutic. The lysosomal cysteine peptidase cathepsin L appears to be critical for SARS-CoV as well as SARS-CoV-2 spike protein activation. As such, this cysteine peptidase is a promising therapeutic target for the development of antiviral drugs. Nevertheless, the contribution of other cysteine cathepsins should not be overlooked. Future work should focus on evaluating the CoV antiviral activity of a number of cathepsin inhibitors developed for treatment of various diseases, and on repurposing their application for treatment of viral infections, but in a way not to compromise the host antiviral immune response.
